# Integrative sRNA, DNA Methylation, and Transcriptomics Reveals Dynamic Epigenetic Reprogramming of *Meloidogyne javanica*-Induced Galls in Arabidopsis

**DOI:** 10.3390/ijms27104365

**Published:** 2026-05-14

**Authors:** Jose Domínguez-Figueroa, Ana Cláudia Silva, Patricia Abril-Urias, Sebastian Y. Müller, Maria Jose Ladera-Carmona, Patrick Schäfer, Victoria Baca-González, Elena Caro, Carolina Escobar

**Affiliations:** 1 Facultad de Ciencias Ambientales y Bioquímica, Universidad de Castilla-La Mancha, E-45071 Toledo, Spain; patricia.abril@uclm.es; 2 Fundación FUNDECYT (Fundación para el Desarrollo de la Ciencia y la Tecnología)—Parque Científico y Tecnológico de Extremadura, Avda. de Elvas s/n, 06006 Badajoz, Spain; 3 Alva Genomics GmbH, Hans-Knöll-Str. 6, 07745 Jena, Germany; seb.mueller@alva-genomics.com; 4 Institute of Phytopathology, Centre for BioSystems, Land Use and Nutrition, Justus Liebig University, Heinrich-Buff-Ring 26, 35392 Giessen, Germany; maria.ladera.carmona@hhu.de (M.J.L.-C.); patrick.schaefer@agrar.uni-giessen.de (P.S.); 5 Centro de Biotecnología y Genómica de Plantas (CBGP), Universidad Politécnica de Madrid and Instituto Nacional de Investigación y Tecnología Agraria y Alimentaria-Consejo Superior de Investigaciones Científicas (UPM-INIA/CSIC), Campus de Montegancedo, 28223 Madrid, Spain; victoria.baca@inia.csic.es (V.B.-G.); elena.caro@upm.es (E.C.); 6 Departamento de Biotecnología-Biología Vegetal, Escuela Técnica Superior de Ingeniería Agronómica, Alimentaria y de Biosistemas, Universidad Politécnica de Madrid (UPM), 28223 Madrid, Spain

**Keywords:** root-knot nematodes, sRNAs, DNA methylation, transcriptomics, rasiRNAs, miRNAs

## Abstract

Root knot nematodes (RKNs) induce galls, containing multinucleated giant cells (GCs) to nourish them. The differentiation of precursor cells to galls/GCs involves extensive cellular reprogramming with multiple layers of regulation. Epigenetic regulation during the early stages of infection indicates that RNA-directed DNA methylation (RdDM) and microRNA-dependent gene silencing contribute to transcriptional and post-transcriptional reprogramming during gall organogenesis. Although later stages of galls/GC development are crucial for nematode life-cycle maintenance, epigenetic reprogramming events remain largely unexplored. An integrative analysis of sRNAs, DNA methylation, and transcriptomic dynamics in galls induced by *Meloidogyne javanica* revealed that enrichment of 24 nt sRNAs represents a gall hallmark across early and late developmental stages. Fewer gall-distinctive sRNAs were detected at mid-to-late stages than at early stages, alongside a pronounced spatial reorganization of rasiRNA accumulation. At early stages, gall-distinctive rasiRNAs preferentially accumulated in pericentromeric retrotransposon-rich regions, whereas, at mid-to-late stages, they predominantly localized to chromosome arms, matching DNA transposons, promoters, and gene bodies. A decline in the regulatory influence of miRNAs was observed as infection progressed, possibly reflecting a transition toward specialized regulatory states associated with gall maintenance. Moreover, three regulatory modules, miR2111-5p/HOLT, miR172/AP2, and miR156/SPL10, were tightly but oppositely regulated at 3 and 14 days post-infection. Furthermore, miR156/SPL10 showed crucial functions during gall formation and/or maintenance, possibly influenced by hormonal cues involving ARF8 among other ARFs. Our results highlight stage-specific patterns involving sRNA dynamics, DNA methylation, and transcriptomic changes underlying nematode feeding site development and maintenance.

## 1. Introduction

Root-knot nematodes (RKN; *Meloidogyne* spp.), are among the most damaging agricultural pathogens, causing severe yield losses worldwide [1]. These obligate endoparasites penetrate plant roots, migrate intercellularly and establish specialized feeding sites within the vascular cylinder, where they trigger the formation of multinucleated giant cells (GCs) within galls [2]. GCs are the sole nutrient source for the nematode, and their development involves extensive cellular reprogramming. This includes mitosis with incomplete cytokinesis, hypertrophy of the surrounding tissues, and profound transcriptional changes [2,3].

Early-stage galls in *Arabidopsis thaliana* and tomato display generalized gene repression. However, the molecular mechanisms underlying this widespread transcriptional reprogramming remain poorly understood [4,5,6,7,8]. This transcriptional control involves multiple layers of regulation, from transcription factors [9] to epigenetic mechanisms. Growing evidence indicates that epigenetic mechanisms, particularly those mediated by noncoding small RNAs (sRNAs) and RNA-directed DNA methylation (RdDM), play a critical role in the transcriptional reprogramming associated with gall organogenesis [8,10,11,12]. Among sRNAs, microRNAs (miRNAs) such as miR390, miR159 and miR172 are essential regulators during the early stages of RKN establishment in Arabidopsis [12]. miR159 regulates *M. incognita*-induced gall formation through early inhibition of MYB33 translation [13], whereas the miR390/TAS3 module represses auxin signaling mediated by auxin responsive factors such as ARF3 in galls induced by *M. javanica* [10]. In addition, miR319 represses TCP4, increasing plant susceptibility to *M. incognita* infection and reducing jasmonic acid levels in galls [14], while miR172 promotes gall and GC formation by repressing the AP2-like transcription factor TOE1 [15]. More recently, miR408 and miR398 have been shown to promote GC formation during RKN infection by modulating copper homeostasis and redox balance downstream of the transcription factor SPL7 [16]. In tomato, miR167 and miR396 regulate feeding site development via ARF8 and sugar allocation to GCs through the GRF8–STP10 module, respectively [17,18]. Specific miRNAs also regulate cyst nematode parasitism in Arabidopsis—i.e., miR396/GRF1/3, miR858/MYB83, and miR827/ubiquitin-E3-ligase-NLA orchestrate syncytium development and parasitism [12]—while miR778 and miR165a modulate epigenetic regulators SUVH5/6 and HD-ZIP III transcription factors, respectively [19,20].

Concurrently, early gall development is marked by the accumulation of 24-nucleotide repeat-associated small interfering RNAs (rasiRNAs), which originate from repetitive genomic regions and are closely associated with the regulation of transposable elements (TEs) and transcriptional silencing through RdDM [10,11].

Despite these advances, major knowledge gaps remain regarding later stages of gall development, during which nematodes must sustain long-term feeding while keeping host defense responses and programmed cell death mechanisms in check. First, the dynamics of sRNA populations during later stages of gall development and their influence on TE regulation are largely unexplored. Second, the relationship between differential sRNA accumulation, DNA methylation, and global transcriptional changes during gall maturation has not been systematically addressed. Third, the functional roles of specific miRNAs and rasiRNAs in shaping gall development are still unclear. Understanding these aspects is essential to uncover how epigenetics, including post-transcriptional mechanisms, orchestrate plant cell reprogramming in response to nematode parasitism.

In this study, we investigated the relationships between sRNAs, transcriptional reprogramming, and DNA methylation during gall development at early and late infection stages (3 and 14 dpi). We aimed to determine whether distinct classes of sRNAs define gall identity across developmental stages and to what extent their spatial distribution is dynamically remodeled during infection. In addition, we explored the regulation of specific miRNA–target modules across gall progression, including miR2111-5p/HOLT, miR172/AP2, and miR156/SPL10, with a particular focus on the role of the miR156/SPL10 module in gall formation and maintenance.

## 2. Results and Discussion

### 2.1. Overview of sRNA Dynamics in Late-Stage Galls

In earlier studies, we described for the first time the differential expression of the sRNA population in early-stage galls (3 dpi) induced by *M. javanica* in Arabidopsis roots as compared to uninfected roots [10]. Here we investigated the dynamic regulation of the sRNA population from early to mid-to-late stages (14 dpi) in galls. Six independent sRNA libraries were generated: three independent biological replicates from hand-dissected galls at 14 dpi and three from corresponding uninfected root segments (see Materials and Methods).

**Figure 1:**
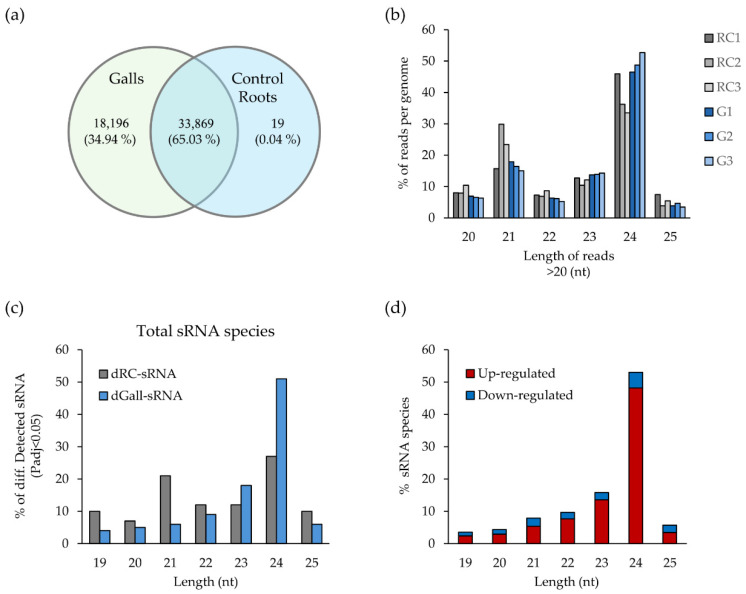
sRNA profiling in Arabidopsis galls induced by *M. javanica* at 14 dpi. (**a**) Venn diagram showing the distribution of sRNA sequences between galls and control roots. (**b**) Length distribution of sRNA clean reads for each of the six libraries prepared from control roots (RC1, RC2, RC3) and galls (G1, G2, G3). (**c**) Percentage of differentially detected sRNA species (Padj < 0.05) classified as distinctive for control roots (dRC-sRNA; only detected in control roots) or galls (dGall-sRNA; only detected in galls). (**d**) Percentage of these sRNA species that are up- or downregulated as compared to control roots in galls at 14 dpi.

At 14 dpi most sRNAs were shared between galls and control roots (65.03%; Figure 1a; Table S2). Gall-distinctive sRNAs (dGall-sRNA; sRNAs present in galls, but not in control roots) accounted for 34.94%, whereas only a small fraction (0.04%) was distinctive to control roots (dRC-sRNAs; present in control roots, but not in galls). These data contrast with early infection stages, when gall-distinctive sRNA sequences were predominant (58%) [10]. The length distribution of clean reads showed that sRNAs of 24 nt, likely corresponding to rasiRNAs, were very abundant in the 14 dpi gall libraries, while typical miRNA lengths of 20 and 21 nt predominated in the control root libraries (Figure 1b). In line with this, 53% of the deregulated gall-distinctive sRNAs (only detected in galls) at 14 dpi were 24 nt in length, and among these, most of them were upregulated (Figure 1c,d). This landscape of sRNA length distribution at 14 dpi resembled that observed during early infection stages, where rasiRNAs were enriched in galls, but miRNAs dominated in control roots.

Therefore, the consistent enrichment of 24 nt sRNAs represents a hallmark of galls across different infection stages and appears to be conserved in other RKN interactions, such as in galls induced by *M. incognita* at 7 and 14 dpi in Arabidopsis [21] and in galls induced by *M. graminicola* at 3 dpi in rice [22].

### 2.2. Distinctive rasiRNA Accumulation Patterns Highlight Dynamic and Stage-Specific Regulatory Landscapes During Gall Development and Maintenance

We previously reported that *M. javanica* infection in Arabidopsis triggers the silencing of transposable elements (TEs) during early gall development, with predominant silencing of class I TEs, retrotransposons, that rely on RNA intermediates and reverse transcriptase activity. Consequently, retrotransposons are the main targets of rasiRNAs in early-stage galls [11]. rasiRNAs play a pivotal role in maintaining genomic stability by preventing uncontrolled activation and proliferation of TEs, primarily by guiding the RNA-directed DNA methylation (RdDM) mechanism [23,24]. We next examined the dynamics of putative TE regulation during gall development in Arabidopsis, with a particular focus on rasiRNA populations in mid- to late-stage galls.

**Figure 2:**
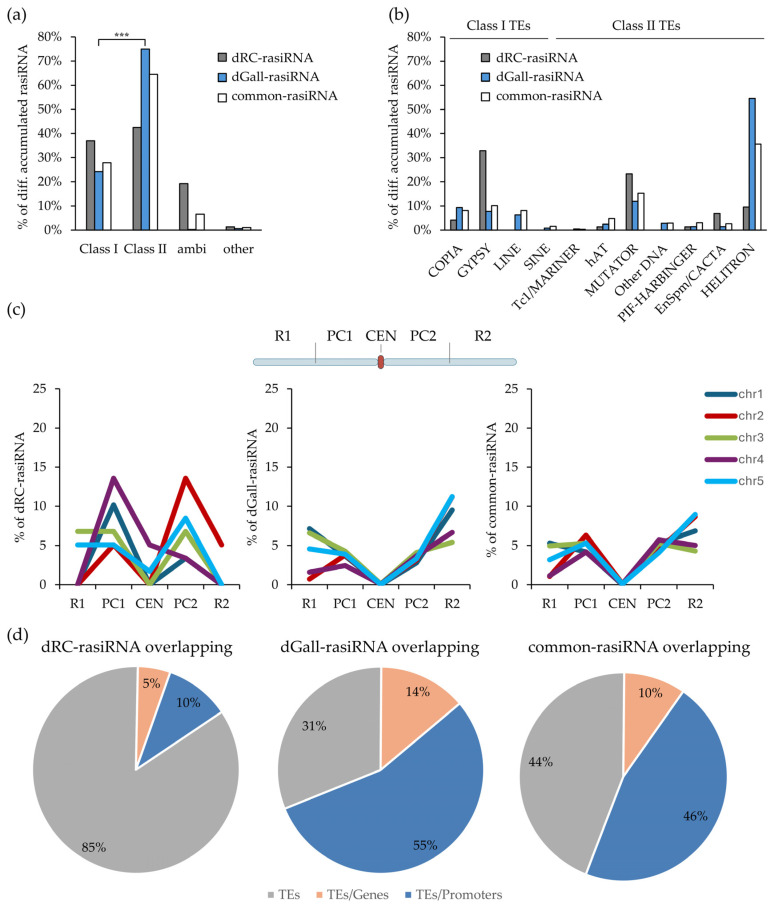
Distinctive accumulation and genomic distribution of rasiRNAs in *M. javanica*-induced galls at 14 dpi. (**a**) Classification of differentially accumulated rasiRNAs according to the type of overlapping repetitive elements: class I (retrotransposons), class II (DNA transposons), ambiguous (rasiRNA-enriched bins with conflicting logFC within a single TE), and others (repeat elements not assigned to known transposon classes). Asterisks (***) indicate a significant overrepresentation of class II-associated rasiRNAs in dGall-rasiRNAs relative to class I, based on chi-square test analysis and adjusted residual analysis (*p* value < 0.001). (**b**) Distribution of rasiRNAs across transposon superfamilies, categorized into class I and class II. (**c**) Spatial distribution of rasiRNAs across Arabidopsis chromosomal regions, shown for dRC-, dGall-, and common rasiRNAs (left to right). The *x*-axis indicates chromosomal segments: R1 (left arm), PC1 (left pericentromeric region), CEN (centromere), PC2 (right pericentromeric region), and R2 (right arm). (**d**) Genomic overlap of rasiRNAs, shown as percentages for each group. Charts show percentages of rasiRNAs overlapping transposable elements (TEs; grey), TEs and genes (light orange), or TEs and promoter regions (blue). dRC-sRNAs (only detected in control roots), dGall-sRNAs (only detected in galls).

Notably, a marked shift in rasiRNA accumulation was observed in mid-to-late-stage galls as compared to early-stage galls. At 14 dpi, the majority of dGall-rasiRNAs (rasiRNAs only found in the gall libraries; 75%) were complementary to class II TEs, DNA transposons, while 24% aligned to class I TEs and 0.58% mapped to other repetitive genomic regions; no reads aligned to TEs with ambiguous classification (Figure 2a; Table S3a). Conversely, dRC-rasiRNAs displayed a contrasting distribution across TE classes. A comparison of TE class composition between dGall- and dRC-rasiRNAs revealed a significant difference (χ^2^ test, *p* < 0.001), with adjusted residuals indicating a relative overrepresentation of class II-associated rasiRNAs in dGall-rasiRNAs and class I-associated rasiRNAs in dRC-rasiRNAs (Figure 2a). In contrast, at 3 dpi, most of the dGall-rasiRNAs targeted class I TEs [11]. These findings suggest a shift in rasiRNA targeting between early and late stages of gall development, specifically in dGall-rasiRNAs, consistent with a stage-dependent pattern.

Our analysis also revealed that 55% of the dGall-rasiRNAs (1588) matched class II TEs from the HELITRON superfamily, whereas only 10% (7) of the dRC-rasiRNAs (distinctive rasiRNAs in control roots) targeted the same superfamily (Figure 2b; Table S3a). Conversely, during early gall formation, class I retrotransposon superfamilies such as GYPSY, COPIA, and LINE were the most enriched among dGall-rasiRNAs [11]. This observation is consistent with a change in the dynamics of rasiRNA populations between early and mid-to-late stages.

These former results on early gall formation were consistent with the spatial distribution of rasiRNAs across Arabidopsis chromosomes (Figure 2c; Table S3b). To assess the chromosomal dynamics of rasiRNA accumulation during gall development, each chromosome was divided into five distinct regions: left arm (R1), left pericentromeric region (PC1), centromeric region (CEN), right pericentromeric region (PC2) and right arm (R2) [11]. In early-stage galls, dGall-rasiRNAs were predominantly enriched in pericentromeric regions [11] coinciding with the preferential localization of retrotransposons in these regions [8]. In contrast, in mid- to late-stage galls, dGall-rasiRNAs displayed a clear shift in chromosomal distribution, accumulating preferentially in the arm regions (Figure 2c). Moreover, a substantial proportion of these rasiRNAs (69%) overlapped not only with annotated DNA transposable elements but also with promoter regions and gene bodies (Figure 2d). Similarly, Medina et al. [21] described that most of the predicted sRNA clusters differentially expressed in *M. incognita*-induced galls at 7 and 14 dpi were located in promoters or gene bodies. Conversely, dRC-rasiRNAs were predominantly distributed in the pericentromeric regions across all five Arabidopsis chromosomes and showed limited overlap with genes or promoters (Figure 2c,d). Common rasiRNAs, on the other hand, were broadly distributed across all chromosome regions except the centromeres (Figure 2c,d).

Together, these observations highlighted a marked spatial reorganization of rasiRNA accumulation in mid- to late-stage galls relative to early gall development, reflecting dynamic and stage-specific regulatory landscapes throughout the course of the infection.

### 2.3. Comprehensive Transcriptomic Analysis of Gall Formation Across Developmental Stages

We aimed to investigate transcriptomic changes during gall formation in *M. javanica*-infected Arabidopsis at mid-to-late infection stages. To this end, we performed RNA-seq analyses of galls induced by *M. javanica* in Arabidopsis at 14 dpi. Additionally, we compared this dataset with previously published early-stage transcriptomic data from galls at 3 dpi obtained under the same experimental conditions and processed using the same bioinformatic pipeline and parameters [8], allowing us to assess temporal expression trends throughout the infection process. This comprehensive dataset also enabled integrative comparisons with other omics approaches described in subsequent sections of the manuscript.

**Figure 3:**
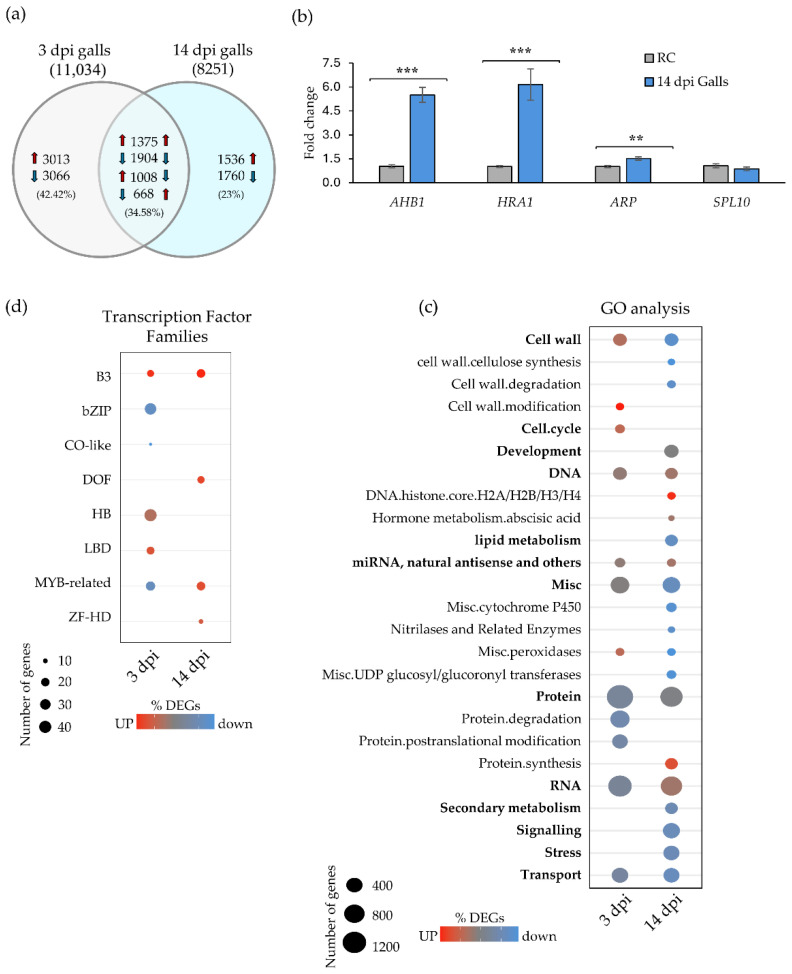
Transcriptional reprogramming during early and mid-to-late stages of *M. javanica*-induced galls in Arabidopsis. (**a**) Venn diagram showing the overlap and direction of differential gene expression in galls at 3 and 14 dpi as compared to control roots. Number of up- and downregulated genes are indicated for each gall stage, along with shared and stage-specific, differentially expressed genes (DEGs), including those with opposite expression patterns between stages. Blue and red arrows indicate downregulated and upregulated genes, respectively. (**b**) Validation of RNA-seq data by qRT-PCR of *AHB1*, *HRA1*, *ARP* and *SPL10* genes. Statistical analyses were performed using a Welch’s *t*-test. Significant differences as compared to control roots (RC) are indicated by asterisks (***, *p* < 0.001; **, *p* < 0.01). Values are presented as mean ± SE. (**c**,**d**) Dotplots showing selected functional categories annotated using MapMan v3.7.1 (**c**) and transcription factor families (**d**) with significantly different expression profiles at 3 and/or 14 dpi (Wilcoxon test, FDR < 0.05). Circle size represents the number of DEGs per category, and circle colour reflects the direction of regulation: red indicates predominance of upregulated genes, blue indicates downregulation, and intermediate tones indicate mixed responses.

A total of 8251 differentially expressed genes (DEGs) were identified in galls at 14 dpi *versus* control root tissue (Table S4; Figure 3a). Notably, a higher number of DEGs (11,034) were detected in galls at 3 dpi [8], representing an overall 25% reduction in DEGs at the later stage (Figure 3a). This decrease was primarily attributed to a 33.7% reduction in upregulated genes (5396 at 3 dpi *versus* 3579 at 14 dpi), whereas the number of downregulated genes declined more moderately by 17.1% (Figure 3a). Further analyses revealed that 6079 DEGs were exclusive to early-stage galls, while 3296 DEGs were exclusive to mid-to-late-stage galls. In addition, 4955 DEGs were shared between both timepoints, including 1375 upregulated and 1904 downregulated genes. Among the DEGs detected at 14 dpi, 56% maintained their expression pattern from 3 dpi, whereas 40% were uniquely DE at the later stage. Interestingly, 1676 DEGs exhibited opposite expression patterns between the two infection stages: 1008 genes were upregulated at 3 dpi but downregulated at 14 dpi, while 668 displayed the reverse trend, being downregulated at 3 dpi and upregulated at 14 dpi (Figure 3a).

To further support the robustness of the transcriptomic dataset at 14 dpi, we validated the expression levels of representative induced genes (*ARABIDOPSIS HEMOGLOBIN*, *AHB1*; *HYPOXIA RESPONSE ATTENUATOR1*, *HRA1*; *SQUAMOSA PROMOTER BINDING PROTEIN-LIKE 10*, *SPL10*, and *ACTIN-RELATED PROTEIN*, *ARP*) by qRT-PCR. Their expression patterns were consistent with the RNA-seq results, as all genes but SPL10 were induced in galls at 14 dpi as compared to uninfected tissues, confirming the reliability of the dataset (Figure 3b).

To investigate key differences and similarities in the biological processes relevant at both infection stages, DEGs were classified by gene ontology using MapMan software v3.7.1 [25]. A Wilcoxon rank test with Benjamini–Hochberg correction was applied to identify functional categories with significantly differential behavior relative to all other categories in each dataset (3 and 14 dpi galls; false discovery rate, FDR < 0.05; Table S5). From these, we selected categories displaying contrasting expression patterns between early and late infection stages. Genes related to cell wall metabolism exhibited dynamic regulation across both stages (Figure 3c; Table S5). In galls at 3 dpi, 69.8% of the genes in the cell wall category were upregulated, and the subcategory of cell wall modification was predominant only at this stage, but not at 14 dpi. In contrast, 84.9% of the cell wall genes were downregulated at 14 dpi, and the two predominant subcategories at this stage, cell wall degradation and cellulose synthesis, showed distinct differential profiles (Figure 3c; Table S5). These patterns suggest a functional transition from active cell wall remodeling during early gall development to structural stabilization during the mid-to-late infection phase. These results suggest that gall maturation may be associated with a general decrease in cell wall metabolic activity, rather than sustained remodeling or increased cellulose production. Consistent with this, previous transcriptomic studies in Arabidopsis giant cells revealed differential expression of genes involved in cell wall remodeling at early infection stages [6]. More recent work has further highlighted the importance of plant cell wall remodeling during nematode establishment, particularly through the activity of cell wall-degrading enzymes such as β-1,4-endoglucanases. In this context, the *CsCEL1* gene in the RKN interaction has been shown to modulate cellulose content and influence host susceptibility during *M. incognita* infection [26].

The cell cycle category showed a significantly differential profile compared to the other categories only at 3 dpi, but not at 14 dpi, with 73.53% of DEGs being upregulated (Figure 3c; Table S5). This pattern is consistent with the pronounced cellular changes occurring during the establishment of feeding sites at early infection stages, characterized by active mitosis with incomplete cytokinesis within the GCs and by proliferation of surrounding tissues within the galls [2]. In addition, the DNA category showed significant differences relative to the other categories at both infection stages. Notably, genes related to histone synthesis were significantly upregulated exclusively in galls at 14 dpi (Figure 3c; Table S5). This likely reflected an increased demand for histone binding at mid-to-late stages to maintain chromatin structure that had suffered multiple rounds of DNA replication, not only due to repeated mitosis with partial cytokinesis, but also to activate endoreduplication as GCs reached their final size [3].

Genes within the *protein* category exhibited distinct expression trends between early- and mid-to-late-stage galls. At 3 dpi, genes associated with protein degradation and post-translational modification pathways were predominantly downregulated and showed a significantly different profile to the rest of the categories (Figure 3c; Table S5). In contrast, at 14 dpi, genes involved in protein synthesis were largely upregulated (Figure 3b; Table S5) suggesting increased translational activity. This shift may reflect the growing nutritional demands of the developing nematode during sustained feeding at later infection stages. Taken together, these transcriptional patterns are consistent with a transition from an early proliferative phase associated with J2 establishment to a later stage characterized by increased metabolic activity required for nematode growth and feeding.

The RNA functional category displayed stage-specific regulation, with notable changes in genes associated with sRNAs, in accordance with the previous sections (Figure 3c; Table S5). These findings underscored the dynamic transcriptional and post-transcriptional adjustments that occur during gall formation. Genes encoding transcriptional regulators (transcription factors; TFs) were predominant in the RNA category (Table S4) and key players in gall and GC formation [9]. Therefore, we analyzed further TF expression patterns in detail.

Galls at 3 dpi showed upregulation of specific TF families (B3 domain, B3; homeobox, HB; lateral organ boundaries, LBD), while bZIP (basic leucine zipper domain) and MYB-related families were repressed; all exhibiting significantly distinct profiles as compared to other TF families (Figure 3d). Notably, *LBD* genes were specifically upregulated at early stages but not at 14 dpi, consistent with their known roles. For example, LBD16 is known to be involved in the early stages of lateral root formation and in early gall/GC development [27]. Downregulation of bZIP members is also characteristic of early infection stages. Supporting this, silencing of *TGA1a*, a member of the bZIP family, in the tomato resistant cultivar Motelle led to increased susceptibility to RKNs [28]. In contrast, MYB-related families were upregulated at 14 dpi, showing the opposite trend compared to 3 dpi galls. Similarly, members of the ZF-HD (Zinc Finger–Homeodomain) and DOF (One Finger DNA binding) families were upregulated and displayed differential expression profiles at 14 dpi, but not during early infection (Figure 3d). Both TF families were therefore characteristic of mid-to-late-stage galls. In accordance, the MYB TF family showed the highest number of members at mid-to-late stages of infection in *M. incognita*-induced galls [4]. However, to date, there is no functional characterization of ZF-HD or DOF TFs in the context of RKN interaction, as there is a limited number of TF families investigated in this pathosystem, as reported by Dominguez-Figueroa et al. [9].

Taken together, the TFs predominant at each gall developmental stage exhibited markedly distinct expression patterns, likely reflecting the underlying biological processes: an initial phase characterized by active cell proliferation, followed by a phase of cell differentiation and expansion during gall maturation. While TFs serve as key regulators of numerous developmental and stress-responsive pathways, epigenetic mechanisms, particularly DNA methylation, also played crucial roles in modulating gene expression during RKN infection in Arabidopsis at early stages [8]. Therefore, we next sought to explore the relationship between stage-specific DNA methylation patterns and gene expression at 14 dpi.

### 2.4. Shaping DNA Methylation During Gall Development and Its Correlation with Expression Patterns

Early-stage galls (3 dpi) exhibited extensive hypermethylation of TEs, predominantly located in pericentromeric regions [8]. This hypermethylation effectively suppressed the activation of retrotransposons (class I TEs), particularly those of the GYPSY superfamily, which were transcriptionally repressed in galls as compared to control roots. This pattern aligned with the preferential location of dGall-rasiRNAs [11]. In contrast, mid-to-late-stage galls displayed pronounced hypomethylation of TEs, particularly those situated in arm regions of chromosomes. Concurrently, hypermethylated regions at this stage were more frequently found in promoters and gene bodies within chromosome arms [8]. This observation was further supported by the accumulation of dGall-rasiRNAs complementary to chromosome arms, with notable enrichment in promoters and genic regions (Figure 2c,d).

In early-stage galls, where host cells undergo extensive transcriptional reprogramming to initiate gall formation, hypermethylation of retrotransposons may function as a protective mechanism to maintain genomic integrity and stability [8]. This is particularly important during the rapid mitotic activity with incomplete cytokinesis that occurs within developing GCs, a distinct process from that occurring at 14 dpi (Figure 3c). By contrast, once galls and GCs are nearly fully developed and mitosis has ceased, around 14 dpi [29], such stringent retrotransposon silencing may no longer be as critical.

We performed a correlation analysis of the differentially methylated DNA regions (DMRs) with that of the DE genes from the RNAseq at 14 dpi and compared it with the same previous analyses performed at 3 dpi [8]. At 3 dpi, 50 DE genes showed correlation between their expression and methylation status, either in their promoters or coding regions, of which 39 corresponded to hypermethylated regions (78%; Table 1 and Table 2). At 14 dpi, 28 DE genes correlated with their methylation state (Table 1 and Table 2). However, in contrast to 3 dpi, only 6 of these genes were associated with hypermethylated regions (21%; Table 1 and Table 2). This observation was consistent with the predominance of hypermethylation in 3 dpi galls, whereas no significant differences in methylation were detected at 14 dpi between galls and non-infected control tissues [8]. Moreover, the number of genes potentially regulated by DNA methylation at early stages was approximately twofold higher than at 14 dpi, further supporting the predominance of epigenetic mechanisms at 3 dpi. In line with this, while DNA methylation is often associated with transcriptional repression, this relationship is context-dependent, as gene body methylation can be compatible with active transcription and promoter methylation is not invariably linked to repression [30,31]. Therefore, correlations between DNA methylation and gene expression should be interpreted with caution.

Among these putatively methylation-regulated genes, no predominant biological categories could be identified; they included genes related to the cell wall, TFs, basic cellular processes, etc. Notably, the only gene that was hypermethylated and downregulated at both infection stages was related to plant defenses, encoding a cysteine/histidine-rich C1 domain family protein which has been reported in pepper to increase susceptibility of *Xanthomonas campestris* pv. *vesicatoria* when silenced, and to enhance resistance to *Hyaloperonospora arabidopsidis* in Arabidopsis when overexpressed [32]. Additionally, an AIG2-like (avirulence-induced gene) family protein was hypermethylated and repressed at 14 dpi, while CYP81D11 (encoding a CYTOCHROME P450, FAMILY 81, SUBFAMILY D, POLYPEPTIDE 11), and CEJ1 (COOPERATIVELY REGULATED BY ETHYLENE AND JASMONATE 1), both associated with plant defense responses, were repressed and differentially methylated at 14 dpi. Interestingly, these two genes were also repressed at 3 dpi but were not differentially methylated, suggesting that distinct regulatory mechanisms may operate at the two infection stages.

In contrast to the correlation observed for hypomethylated gene promoters at 3 dpi and upregulation of plant defense genes at 7 dpi in *M. graminicola*-induced galls in rice [33], we did not observe this tendency in our biological system, except for one gene related to abiotic heat stress; *HTT3* (Table 2) was hypomethylated in its promoter region and induced at 14 dpi.

### 2.5. Global Overview of miRNA-Mediated Gene Regulation Across Early and Mid-to-Late Infection Stages of Root-Knot Nematodes

**Figure 4:**
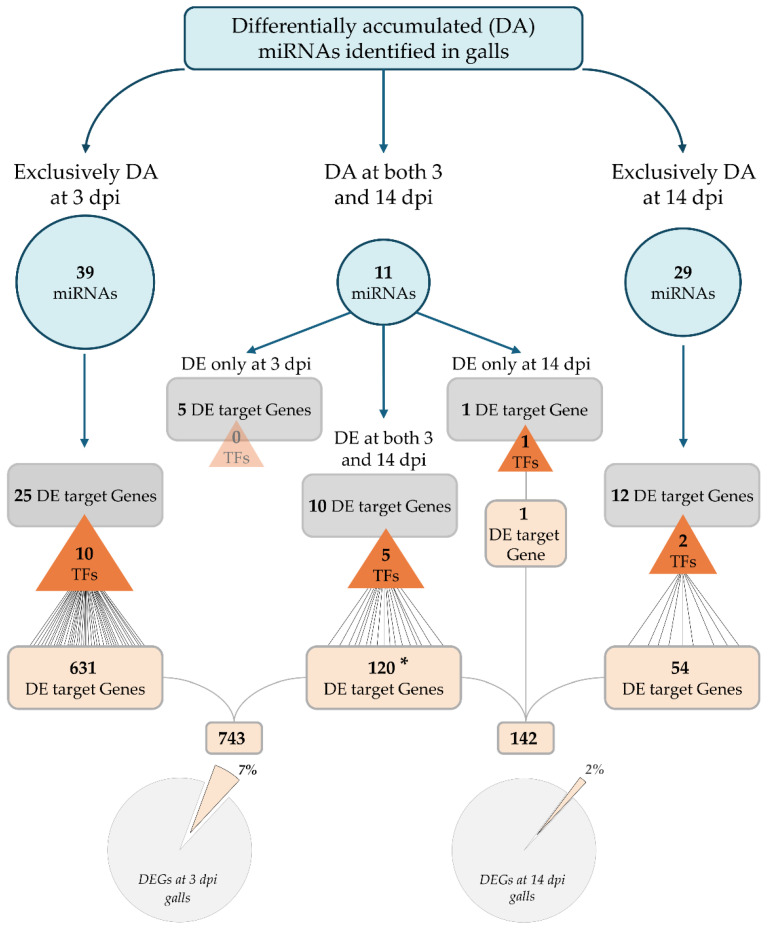
Schematic overview of miRNA-mediated transcriptional regulatory cascades during RKN gall development. Differentially accumulated (DA) miRNAs at 3 and 14 dpi were classified as stage-specific or shared between infection stages (blue circles). Corresponding miRNA putative target genes that are also differentially expressed (DE) at each gall stage are shown (grey rectangles), with transcription factors (TFs) highlighted (orange triangles). Putative targets of miRNA-regulated TFs that are DE in galls are represented by beige rectangles, illustrating hierarchical transcriptional regulatory networks. Asterisk indicates that, among the 120 DEGs downstream of miRNA-regulated TFs present at both stages, 78 display consistent expression trends across the gall development. The non-redundant total of DE target genes yields 743 and 142 DE target genes at 3 and 14 dpi galls, respectively, representing 7% and 2% of all DEGs at these stages, as indicated in the pie chart.

To evaluate the putative impact on gene regulation of miRNAs during gall development, we integrated the expression profiles of differentially accumulated (DA) miRNAs with those of DEGs in galls at early and mid-to-late stages. A total of 39 miRNAs were uniquely DA at 3 dpi, putatively targeting 25 DEGs, among which 10 encoded transcription factors (TFs; Figure 4; Table S7). These TFs were predicted to regulate 631 downstream DEGs, revealing a broad hierarchical amplification of miRNA-mediated post-transcriptional control during early gall formation. In contrast, 29 miRNAs were exclusively DA at 14 dpi, putatively targeting 12 DEGs, of which only two were TFs (Figure 4; Tables S6 and S7). These TFs were predicted to regulate 54 DEGs, suggesting a more restricted miRNA/TF regulatory cascade at mid-to-late stages compared with early stages.

A subset of 11 DA miRNAs was shared between both infection stages. These miRNAs were predicted to regulate five DEGs exclusively at 3 dpi and one at 14 dpi, the latter encoding a TF predicted to regulate one DE target gene. Ten putative target genes were common to both stages, five of which were TFs. These TFs were predicted to regulate 120 downstream DEGs, including 78 exhibiting consistent expression trends across stages, suggesting partial conservation of miRNA-dependent regulatory modules throughout gall development (Figure 4). When considering all non-redundant DEGs targeted by TFs under miRNA control, we predicted that miRNA-mediated regulation accounted for approximately 7% of total DEGs at 3 dpi but only 2% at 14 dpi. This integrative analysis revealed a decline in the regulatory influence of miRNAs as infection progressed (Figure 4).

Together, these results revealed a pronounced stage-dependent shift in the regulatory architecture of miRNA activity during gall development. While early gall formation relied on extensive and multilayered miRNA/TF regulatory networks that putatively drive large-scale transcriptional and post-transcriptional reprogramming, mid-to-late-stage galls exhibited a reduction in these networks, reflecting a transition toward more stable and specialized regulatory states possibly associated with gall maintenance.

### 2.6. Differential Accumulation of miRNAs During Early and Mid-to-Late Stages of Infection

To characterize the dynamics of miRNA regulation during gall development, we analyzed their accumulation profiles in Arabidopsis during *M. javanica* infection. At 14 dpi, a total of 184 miRNAs were detected in at least one of the six libraries analyzed: 168 were present in both galls and control roots, 9 were detected only in galls, and 7 only in control root libraries (Table S6). Similar numbers were observed in galls at 3 dpi [10]. Among the DA miRNAs, 11 and 13 showed higher accumulation in galls as compared to control roots at 3 dpi and 14 dpi, respectively (from now on, we will refer to them as induced; Table 3a–c), whereas 44 and 32 showed lower accumulation in galls at 3 and 14 dpi, respectively (from now on, we will refer to them as repressed; Table 3a–c). Notably, only 11 miRNAs were DA at both infection stages, and all of them were repressed, while none were induced at both stages (Table 3c). Interestingly, five miRNAs showed opposite accumulation patterns at early and mid-to-late infection stages (Table 3c). Thus, DA miRNAs in galls as compared to their control roots were markedly distinct at early and late stages of infection.

**Figure 5:**
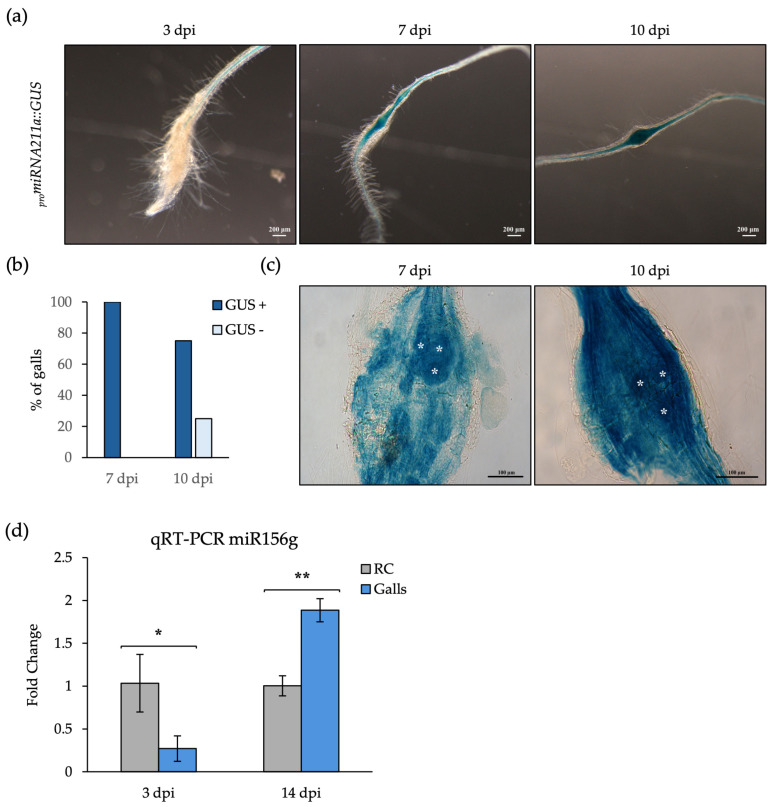
Validation of miR2111a and miR156g dynamics and target gene expression during gall development. (**a**) Representative galls of the line *promiR2111a::GUS* at 3, 7, and 10 days post-infection (dpi). Scale bar: 200 µm. (**b**) Proportion of galls displaying detectable *versus* undetectable GUS staining. Thirty galls were analyzed per time point. (**c**) Tissue clarification with BABB revealed GUS signal localized within giant cells (white asterisk) at 7 and 10 dpi. Scale bar: 100 µm (**d**) Relative accumulation of miR156g at 3 and 14 dpi, validated by qRT-PCR. Asterisks indicate statistically significant differences (*, *p* < 0.05; **, *p* < 0.01).

Among the miRNAs showing opposite regulation at both infection stages, we validated the accumulation of miR2111a and miR156g. The *promiR2111a::GUS* line showed signal in the lateral root primordia of uninfected roots (Figure S1), as previously described [34]. A clear GUS signal in the center of galls at 7–10 dpi was also observed, whereas no signal was detected at 3 dpi (Figure 5a,b). After tissue clarification, the signal was clearly visible in GCs (Figure 5c). These observations indicated that the miR2111a promoter is activated at mid-to-late infection stages, consistent with its accumulation pattern, since promoter activation is expected to precede miRNA accumulation (Table 3; Figure 5a–c). In this context, the described miR2111-5p target gene, *TML*/*HOLT* (*TOO MUCH LOVE*/*HOMOLOGUE OF LEGUME TML*) [34], which encodes an F-box Kelch repeat protein, was upregulated in galls at 3 dpi according to RNA-seq [8] data but did not show differential expression at 14 dpi (Tables S4 and S7). The miR2111-TML regulatory module controls nodulation during symbiotic bacterial interactions and, interestingly, this regulon is conserved in *Arabidopsis thaliana*, a non-symbiotic plant, where it contributes to the regulation of lateral root (LR) formation under different nitrogen conditions [34]. Since signaling pathways involved in LR formation are also essential for gall formation [27,35], our data suggest a potential role for the miR2111-5p/HOLT interaction during gall organogenesis, although further functional analyses will be required to elucidate its direct regulatory role.

In parallel, the accumulation pattern of the mature miRNA156g, repressed in galls at 3 dpi and induced in galls at 14 dpi as indicated by the sRNA-seq data, was confirmed by qRT-PCR (Figure 5d; Table 3). Notably, the target genes of miR156g encoded TFs of the SQUAMOSA (SBP/SPL) family, such as SPL9 and 10 [36]. Both genes were induced at early-gall developmental stages [8] and were not differentially expressed at mid-to-late stages as compared to uninfected control roots, a trend consistently observed in the RNA-seq dataset and validated by qRT-PCR for *SPL10* (Table S7; Figure 5d). As expected for a canonical miRNA-target relationship, their expression patterns were opposite to the miR156g accumulation profile.

Furthermore, the antagonistic regulation between miR156 and miR172 constitutes a well-established mechanism controlling developmental timing and phase transition in plants. In this regulatory module, miR172 promotes flowering, and thus new organ formation, through the repression of AP2-like TFs, ultimately enabling the activation of FLOWERING LOCUS T (FT). Conversely, miR156 maintains juvenile traits by repressing SPL TFs [37]. Interestingly, SPL9 and 10 redundantly promoted the transcription of miR172 genes [37]. A similar opposite regulatory pattern of both miRNAs was observed during gall development: miR172 was induced at early stages [15] and repressed at 14 dpi, whereas miR156g was repressed at 3 dpi and induced at 14 dpi (Table 3). This is consistent with previous studies, where miR172 has been shown to play a crucial role during early gall development by repressing an AP2-like transcription factor, TARGET OF EARLY ACTIVATION TAGGED 1 (TOE1), thereby allowing the activation of FT [15], a regulatory module also implicated in tuberization and nodulation [38,39,40].

In addition to the miR156/miR172 module, the remaining members of the five miRNAs exhibiting opposite accumulation patterns between early- and late-stage galls (Table 3c), also displayed dynamic, stage-specific regulations. Among these, miR390a and miR391 were induced in galls at 3 dpi but repressed at 14 dpi. The target genes of miR390a-5p, AL6 and AL7 were not DE in early-stage galls [8] but were induced in galls at 14 dpi according to RNA-seq data (Table S7). These genes encode Alfin-Like proteins that bind to di- or tri-methylated histone H3 (H3K4me3/2) [41], thereby playing a role in epigenetic regulation via histone remodeling, processes that change also dynamically during RKN infection [42].

Interestingly, 11 mRNAs were repressed throughout gall development at early and late stages (Table 3c). Among the described target genes upregulated in galls at 3 and 14 dpi, we found several TFs from the HD-ZIP class III family, such as REV, PHB, PHV and CNA (*REVOLUTA*, *PHABULOSA*, *PHAVOLUTA* and *CORONA*, respectively; Table S7), key regulators of meristem development, organ polarity and vascular formation in Arabidopsis [43]. The expression of these TFs in roots is regulated by miR165/166 [44] and both were found to be repressed in galls at 3 and 14 dpi (Table 3c). miR165/166 control xylem cell type differentiation by degrading HD-ZIP III transcription factor family mRNAs [44]. Some of them as PHB, and likely PHV in roots, are critical for metaxylem specification and/or vascular patterning [44]. This is consistent with previously described processes occurring during RKN infection, which induces extensive vascular remodeling [45,46].

It is worth noting that among the 30 miRNAs exclusively repressed at early-stage galls but not DE at 14 dpi, the miR169 and miR156 families were highly represented, with 11 and 6 members, respectively (miR169d-n and miR156a/c-f/j; Table 3a). Several validated targets of miR169d-n, belonging to the NF-YA Transcription factor family, namely NF-YA3, NF-YA8 and NF-YA9, as well as members of the previously identified SBP family, including SPL9 and SPL10, are known targets of miR156a/c/d/e/f, and, as mentioned before, were upregulated at 3 dpi [8] (Table S7). These TFs are key components of the miR169/NF-YA and miR156/SPL regulons, which promote developmental reprogramming and cell fate transitions during embryogenesis [47]. Additionally, cyclin B2;2 (CYCB2;2), a classical regulator of M-phase entry and a predicted target of miR156a/c/d/e/f, was upregulated in 3 dpi galls [8], consistent with the enhanced mitotic activity observed in GCs during early infection stages [29]. Altogether, these results suggested that the repression of specific miRNAs at early infection stages allowed the induction of their target genes involved in developmental processes and cell growth, as key processes during gall formation [2] (Table S7). In contrast, those identified miRNAs were not DA at 14 dpi (Table 3a) and their putative target genes were also not DE at this stage (Table S7). This finding aligned with the fact that gall organogenesis is nearly completed by 14 dpi.

In conclusion, the accumulation patterns of most miRNAs were consistent with the expression profiles of their putative target genes at 3 and 14 dpi. However, not all targets followed this pattern, supporting the involvement of additional regulatory mechanisms.

### 2.7. Functional Role of miR156-Regulated Developmental Pathways During Infection

**Figure 6:**
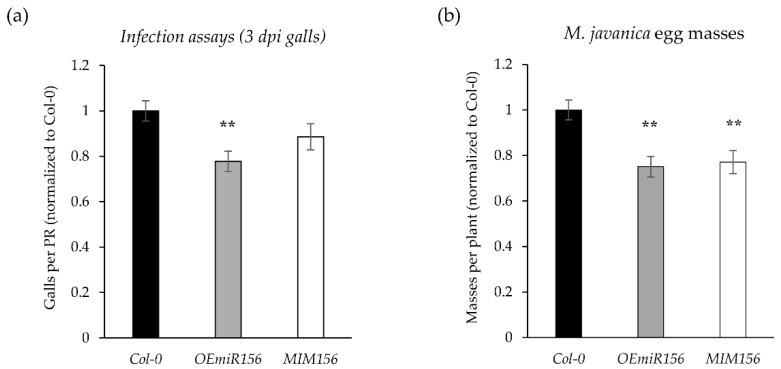
Effects of miR156 deregulation on the infection of *Arabidopsis thaliana* by *M. javanica. In vitro* infection assays were performed comparing the miR156 overexpression line (*OEmiR156*) and the miR156 knockdown line (*MIM156*) with the Col-0 ecotype at 3 days post-infection (dpi). (**a**) The number of galls per primary root (PR), and (**b**) the number of egg masses per plant at 45 dpi normalized to that of Col-0, is represented. Statistical analyses were performed using six independent experiments per line (*n* = 100 plants per replicate and line) using a *t*-test. Significant differences as compared to Col-0 are indicated by asterisks (**, *p* < 0.01). Values are presented as mean ± SE.

Based on the observed tight regulation of miR156g at early and mid-to-late stages, together with its opposite expression pattern relative to miR172, we hypothesized that miR156g may play an important functional role during RKN parasitism. To test this hypothesis, Arabidopsis plants of a *miR156* overexpression line (*OEmiR156*) and a *miR156* knockdown line (*MIM156*) were inoculated with *M. javanica*. At 3 dpi, only the *OEmiR156* line exhibited a significant reduction in the number of galls per plant as compared to Col-0 control plants (Figure 6a). However, at 45 dpi, both transgenic lines displayed a significantly lower number of egg masses per plant relative to the control, indicating impaired nematode reproduction (Figure 6b). These findings aligned with the sRNA-seq data showing a dynamic regulation of miR156 during infection, initial downregulation at 3 dpi followed by an upregulation at 14 dpi, and indicated that *miR156* overexpression (*OEmiR156*) at early infection stages negatively affects nematode establishment, whereas repression of miR156 (*MIM156* line) significantly impairs *M. javanica* reproduction. In agreement with the reduced gall formation, the *OEmiR156* line also showed a lower reproduction rate as compared to the control (Figure 6a,b). Together, these results suggest that precise temporal control of miR156 expression is critical for successful RKN establishment and gall maintenance.

**Figure 7:**
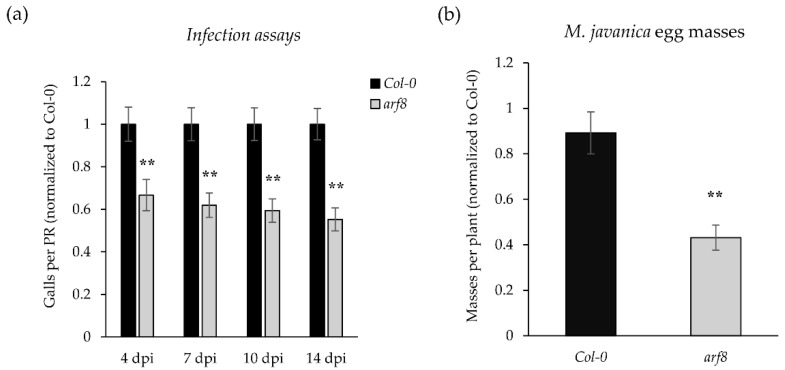
Effects of ARF8 loss of function on *Arabidopsis thaliana* infection by *M. javanica*. *In vitro* infection assays were performed comparing the knock-out mutant *arf8-7* with the Col-0 control at 4, 7, 10, and 14 dpi. (**a**) Number of galls per primary root (PR) and (**b**) number of egg masses per plant at 45 dpi after normalization to Col-0 are shown. Statistical analyses were performed across three independent experiments (*n* = 100 plants per replicate) using a *t*-test. Significant differences relative to Col-0 are indicated by asterisks (**, *p* < 0.01). Values are presented as mean ± SE.

Interestingly, SPL9 and SPL10 negatively regulate lateral root (LR) formation by modulating auxin-responsive pathways [48], and auxin signaling is crucial during gall/GC formation [27]. In this respect, ARF6 and 8 are known to regulate *MIR172c* binding to its promoter and to act as positive regulators of miR172c, linking auxin signaling to the development of reproductive organs during fruit formation in Arabidopsis [49]. In addition, *MIR172c* is regulated by auxins in early galls [15]. In this respect, we observed from the RNA-seq that *ARF8* was upregulated at 3 dpi [8] when miR172 was transcriptionally active [15] but, in turn, not differentially expressed at 14 dpi (Table S4) when miR172c is downregulated and miR156 is upregulated (Table 3). Therefore, we aimed to analyze the putative role of ARF8 during gall formation. The mutant line *arf8* showed a significant reduction in the number of galls and egg masses (Figure 7a,b) as compared to the control Col-0. All these data point to a tight regulation of the miR156/SPL10 and miR172/AP2 modules during gall formation, possibly influenced by hormonal cues involving ARF8 among other ARFs. In this respect, CRISPR/Cas9-generated *arf8a*, *arf8b*, and *arf8ab* mutant lines of ARF8 homologues in tomato displayed reduced gall numbers and reproductive parameters, together with a reduced GC area [17], supporting a conserved and major role for ARF8 during gall development.

It should be noted that all statements made throughout the manuscript refer to galls, which are heterogeneous tissues containing the GCs; therefore, GC-specific signatures might be diluted in our analysis.

## 3. Materials and Methods

### 3.1. Plant Material and Nematode Population

The Columbia (Col-0) ecotype of *Arabidopsis thaliana* was used as the wild-type control and as the genetic background for all transgenic lines. The following, previously described lines were included in this study: the *miR156* overexpression line (*OEmiR156*) [36], the miR156 target mimicry line (*MIM156*) [50], the *_pro_miR2111::GUS* reporter line [34] and loss-of-function mutant *arf8-7* (GK-510C01-019520) [51]. These Arabidopsis transgenic lines were used for *Meloidogyne javanica* infection assays and gene expression analyses.

*M. javanica* was propagated *in vitro* on etiolated cucumber (*Cucumis sativus*) roots, and egg masses were collected and incubated in sterile tap water at 26 °C to stimulate hatching, as previously described [8].

### 3.2. Plant–Nematode Infection Assays

Arabidopsis seeds were surface-sterilized, and plants were grown vertically on Gamborg B5 agar plates under controlled environmental conditions (16 h light/8 h dark, 23 °C). Five-day-old seedlings were inoculated with second-stage juveniles (J2s) of *M. javanica* following a previously described protocol [35]. For infection assays, three to five independent *in vitro* infection tests, with at least 130 plants per line, were performed. The total number of galls was quantified at the selected infection stages. For the reproduction test, the number of egg masses developed was quantified at 45 days post infection (dpi). For histochemical analysis of *_pro_miR2111::GUS*, at least three independent experiments were conducted, and the GUS staining was performed following a previously described protocol [27].

### 3.3. RNA Extraction and Purification

Total RNA was extracted and purified from hand-dissected galls and equivalent control root segments using the AllPrep^®^ DNA/RNA/miRNA Universal Kit (QIAGEN GmbH, Hilden, Germany) following the protocol described in Silva et al. [52]. The RNA samples were quantified and quality-checked using an Agilent^®^ 2100 Bioanalyzer (Agilent^®^ Technologies, Inc., Santa Clara, CA, USA).

### 3.4. RNA-Seq and sRNA-Seq Library Preparation and Bioinformatic Analysis

mRNA and sRNA libraries were prepared from galls and uninfected control tissues 14 days after infection (dpi) collected following a previously described procedure [8]. These samples were obtained from three independent experiments, each including three biological replicates of approximately 300 galls (14 dpi) and three replicates of 500 equivalent uninfected root segments.

mRNA-seq libraries were generated using the TruSeq Stranded mRNA Library Prep Kit (Illumina, Inc., San Diego, CA, USA). Samples were dual-indexed to enable post-sequencing demultiplexing and their libraries were pooled in equimolar amounts prior to sequencing on an Illumina^®^ HiSeq X platform with paired-end 150 bp reads at Macrogen Inc. (Seoul, Republic of Korea). For the comparison between 3 and 14 dpi transcriptomic datasets, raw RNA-seq reads from both time points were processed using the same bioinformatic pipeline and parameters as those applied to the 3 dpi dataset following a previously described procedure [8], ensuring full comparability between developmental stages.

For sRNA-seq, libraries were constructed using Illumina-Solexa Sequencing-By-Synthesis technology at the same facility.

Raw sequencing reads from all libraries were subjected to quality control using FastQC v0.11.9. Adapter trimming and filtering were performed using Cutadapt, with dataset-specific parameters and additional processing steps described below. Post-trimming quality assessment was conducted using FastQC, and MultiQC v1.24.1 was used to summarize quality metrics across samples.

#### 3.4.1. Reference Genome

A composite reference genome consisting of *Arabidopsis thaliana* (TAIR10) and *Meloidogyne javanica* genomes was constructed as a reference, which allowed the separation of reads between the two organisms and the performance of separate investigations.

#### 3.4.2. RNA-seq Data Processing and Analysis

For RNA-seq analysis, adapter sequences were removed using Cutadapt v3.4 with the universal Illumina adapter sequence (AGATCGGAAGAGC) trimmed from both forward and reverse reads. Trimming was performed with a minimum overlap of 1 bp (-O 1) and reads shorter than 20 bp after trimming were discarded (--minimum-length 20). Adapter trimming was executed using 12 threads for computational efficiency.

Reads were aligned to the composite reference genome using STAR aligner v2.7.10b. A STAR genome index was generated with a genomeSAindexNbases parameter of 13, using the Araport11 GTF annotation. Paired-end reads were aligned using 12 threads with the following parameters: a maximum of seven mismatches per read pair (--outFilterMismatchNmax 7) and a maximum of five multimapping positions (--outFilterMultimapNmax 5). Alignments were output as coordinate-sorted BAM files (--outSAMtype BAM SortedByCoordinate). BAM files were indexed using SAMtools.

Read counts per gene were obtained using HTSeq-count v0.13.5 with position-sorted BAM files (-r pos) and strand-specific counting for reverse-stranded libraries (-s reverse). Read assignments were performed against the Araport11 GTF annotation to generate raw count matrices for downstream differential expression analysis.

Differential expression (DE) between galls and control roots was performed using the R/bioconductor package edgeR, first running “glmFit” to fit genewise negative binomial and then “glmLRT”, which conducts likelihood ratio tests. TMM normalization method was used for “calcNormFactors” library normalization. Multiple test correction was carried out by calculating the false discovery rate (FDR) from *p*-values using the Benjamini–Hochberg method as implemented in the R function “p.adjust”. Significance was determined based on FDR < 0.05. Differential expression was expressed as log_2_ fold change (FC) between galls and control roots. Gene annotations and details of the differentially expressed genes (DEGs) are provided in Table S1. Functional categorization of DEGs was performed using MapMan software v3.7.1, which enabled the visualization and assignment of genes to specific biological pathways and processes.

#### 3.4.3. sRNA-seq Data Processing and Analysis

Small RNA sequencing data processing and analysis were performed using a snakemake pipeline (https://github.com/seb-mueller/snakemake_sRNAseq; accessed on 30 June 2023; commit: 1b381a9). Low-quality reads, reads containing 5′ primer contaminants, lacking a 3′ primer, without the insert tag, with poly-A stretches, or shorter than 18 nt were discarded. Resulting small RNA reads (fastq format) were subjected to 3′ adaptor removal (trimming) using cutadapt v3.1 removing Illumina universal adapters. Remaining sequences were mapped to the reference genome of *A. thaliana* (TAIR10).

Mapping was performed using Bowtie version 1.2 with two different approaches. Firstly, multi mapping reads using the parameters “bowtie --wrapper basic-0 -v 0 -k 1 -m 50--best -q” which retain reads mapping to up to 50 locations, assigning each read to its single best-scoring genomic position (ties resolved arbitrarily by index order). Secondly, only uniquely mapping reads were retained using “bowtie –wrapper basic-0 -v 0 -k 1 -m 1 --best -q.” Both were performed requiring 0 mismatches (-v 0). For both approaches, mapped sRNA reads were counted separately for each library based on overlapping bins as defined above. An additional read counting was also performed for each small RNA size class (20–25 nt length) for each bin for a more detailed analysis.

TE class and superfamily distribution analyses were performed using the multi-mapping dataset (m = 50), as TE-derived sRNAs are inherently repetitive and would be severely underrepresented under unique-mapping constraints. In contrast, DESL identification and chromosomal distribution analyses were performed on uniquely mapping reads (m = 1) to ensure locus specificity.

Comparisons of TE class composition between dGall- and dRC-rasiRNAs were performed using a chi-square (χ^2^) test based on the counts of rasiRNAs assigned to TE classes. Adjusted residuals were used to identify TE classes contributing to significant differences.

Two complementary analytical strategies were applied for sRNA analysis, a species approach, in which all individual species (sequenced small RNA with the exact same sequence) were counted across libraries to identify highly expressed individual species such as miRNAs; and a bin/loci approach, where the genome was segmented in adjacent bins of 200 bp to capture the distribution of small RNAs across genomic regions, particularly suitable for rasiRNAs. This bin-based approach captures sRNA abundance at the level of genomic regions, rather than per individual TE copy. The counts per bin or per species were then subjected to standard methods for differential expression (DE) analysis.

DE between galls and control roots was performed using the R/bioconductor package edgeR, as described above for RNA-seq data, with genomic bins used as test intervals.

Analogously to RNAseq nomenclature, instead of DEG we used the term differentially expressed small RNA locus (DESL) to indicate bins (loci) that exhibit statistical differences in counts between conditions. Bins were grouped based on FDR threshold into non-DESL (FDR > 0.9), (0.05 < FDR < 0.9) and DESL (FDR < 0.05). DESL were further classed into +DESL (Gall > RC) and −DESL (Gall < RC). Bins with a total raw count ≤ 10 across all six libraries were excluded prior to differential analysis.

Differential accumulation was expressed as log_2_ fold change (FC) between galls and control roots. Additional details of the differentially accumulated sRNAs are provided in Table S2.

#### 3.4.4. Data Deposition

The complete raw sequencing data have been deposited in the GEO database under accession number GSE320192 (RNA-seq 14 dpi) and GSE320193 (sRNA-seq 14 dpi).

### 3.5. qPCR Validation

For qRT-PCR validation of mRNA-seq results, three independent biological replicates of 14 dpi galls and uninfected root segments as controls were collected. Each replicate consisted of approximately 50 galls or 100 control root segments. A total of 150 ng of RNA per sample was reverse-transcribed using a High-Capacity cDNA Reverse Transcription Kit (Thermo Fisher Scientific, Waltham, MA, USA). Relative transcript abundance was quantified by qRT-PCR with gene-specific primers on a LightCycler^®^ 480 II system (Roche, Indianapolis, IN, USA). The *E2 ubiquitin-conjugating enzyme 21* gene (*UBC-21*) was used as an internal reference. Expression levels in galls were expressed relative to the corresponding control root segments. Each gene was analyzed in three technical replicates per independent biological replicate. Sequences of primers used are listed in Table S1.

For the validation of miR156g accumulation at 3 and 14 dpi, expression levels were analyzed by qRT-PCR using TaqMan^®^ small RNA assays. RNA was obtained from three independent biological replicates of galls and equivalent control root segments at both time points, as described in previous sections. For each reaction, 10 ng of total RNA was reverse-transcribed using the MicroRNA Reverse Transcription Kit (Applied Biosystems, Foster City, CA, USA) in a final volume of 5 µL. Independent reverse transcription reactions were performed for the specific probe aly-miR156g (Assay ID: 005694_mat; Applied Biosystems) and for the reference snoR101 (predesigned TaqMan^®^ small RNA control assays; Applied Biosystems). qRT-PCR reactions were performed with TaqMan^®^ Fast Universal PCR Master Mix, and each cDNA template was analyzed in triplicate.

Relative expression levels were calculated using the ΔΔCt method. Statistical significance was assessed using a two-tailed Welch’s *t*-test on ΔCt values, with adjusted *p* < 0.05 considered significant.

## 4. Conclusions

Our analysis revealed that the enrichment of 24 nt sRNAs is a characteristic of galls across different infection stages. However, fewer gall-distinctive sRNAs were detected at mid-to-late stages as compared to early stages of gall development. Notably, the spatial reorganization of rasiRNA accumulation reflected dynamic and stage-specific regulatory landscapes throughout gall development. At early stages, gall-distinctive rasiRNAs preferentially accumulated in pericentromeric retrotransposon-rich regions, whereas at mid-to-late stages they predominantly localized to chromosome arms, matching DNA transposons, promoters and gene bodies. In parallel, a pronounced stage-dependent shift in the regulatory architecture of miRNA activity during gall development was observed as infection progressed, possibly reflecting a transition toward more stable and specialized regulatory states associated with gall maintenance. In this context, the miR156/SPL10 module, functionally supported in this study, together with the previously described miR172/AP2 at early stages, and the miR2111-5p/HOLT module, exhibited contrasting expression patterns at 3 and 14 dpi. The miR156/SPL10 and miR172/AP2 modules are likely to play crucial roles during gall formation and/or maintenance, potentially modulated by hormonal cues involving ARF8 among other ARFs. The miR2111-5p/HOLT may also contribute to these processes but requires further functional validation. Together, these results highlight dynamic, stage-specific changes in sRNA-mediated regulation, DNA methylation patterns, and transcriptomic profiles that support the formation and maintenance of RKN feeding sites (galls).

These findings provide a framework for further exploration of the role of epigenetic regulation in plant–nematode interactions. In particular, future studies should aim to characterize stage-specific regulatory mechanisms involving sRNAs, DNA methylation, and transcriptional responses. Such approaches may also provide broader insights into epigenetic mechanisms that regulate plant developmental plasticity and responses to biotic stress, with potential implications for crop improvement and sustainable plant protection.

## Figures and Tables

**Table 1:** Correlation of DNA methylation changes with differential gene expression in *M. javanica*-induced Arabidopsis galls at early and mid-to-late stages for genes overlapping differentially methylated regions (DMRs). Data from galls at 3 dpi, as previously published in Silva et al. [8], were complemented with new data of methylation (methylation difference > 15%) and expression data from galls at 14 dpi to highlight the temporal dynamics of gene-associated methylation during gall development. For each overlapping gene, the methylation context (CG, CHG or CHH), direction of methylation change (hyper- or hypomethylation), and corresponding gene expression change (log_2_ fold change) are indicated. Red indicates hypermethylation and upregulated expression; blue indicates hypomethylation and downregulated expression.

				At 3 dpi Galls		At 14 dpi Galls	
Genomic Region	Gene ID	Description	Methylation Context	Difference of Methylation	log_2_FC	Difference of Methylation	log_2_FC
Gene	AT1G66340	ETHYLENE RESPONSE 1 (ETR1)	CG	15	−0.32		
	AT1G73390	Endosomal targeting BRO1-like domain-containing protein	CG	36	−0.4		
	AT2G02090	CHROMATIN REMODELING 19 (CHR19)	CG	23	−0.42		0.27
	AT3G24518	Natural antisense transcript overlaps with AT3G24520	CG	25	−1.51		
	AT3G45840	Cysteine/Histidine-rich C1 domain family protein	CG	20	−1.28	18	−1.92
	AT2G20980	MINICHROMOSOME MAINTENANCE 10 (MCM10)	CG	21	1.05		
	AT3G43600	ALDEHYDE OXIDASE 2 (AAO2)	CG	31	0.3		−0.45
	AT3G45240	GEMINIVIRUS REP INTERACTING KINASE 1 (GRIK1)	CG	37	0.31		
	AT3G60415	Phosphoglycerate mutase family protein	CHH	19	5.12		
	AT4G08400	EXTENSIN 7 (EXT7)	CHG	16	3.22		
	AT4G14760	Kinase interacting (KIP1-like) family protein (NETWORKED 1B, NET1B)	CG	24	0.53		−0.81
	AT5G06195	Novel transcribed region	CG	36	1.74		
	AT5G46290	3-KETOACYL-ACYL CARRIER PROTEIN SYNTHASE I (KASI)	CG	20	0.41		−0.81
	AT3G02920	REPLICATION PROTEIN A 2B, RPA2B	CG	−25	1.13		
	AT3G52680	F-box/RNI-like/FBD-like domain-containing protein	CG			−17	0.56
	AT5G23120	HIGH CHLOROPHYLL FLUORESCENCE 136 (HCF136)	CHG	−22	−1.24		0.56
	AT5G62190	DEAD/DEAH box RNA helicase (PRH75)	CG	−16	−0.61		

**Table 2:** Correlation between gene promoter methylation changes and gene expression in *M. javanica*-induced Arabidopsis galls at 3 and 14 dpi. For each gene, whose promoter overlaps a differentially methylated region (DMR), the methylation context (CG, CHG or CHH), direction of methylation change (hyper- or hypomethylation), and corresponding gene expression change (log_2_ fold change) are indicated. Red indicates hypermethylation and upregulated expression, and blue indicates hypomethylation and downregulated expression.

				At 3 dpi Galls		At 14 dpi Galls	
Genomic Region	Gene ID	Description	Methylation Context	Difference of Methylation	log_2_FC	Difference of Methylation	log_2_FC
Promoter	AT2G31070	TCP DOMAIN PROTEIN 10 (TCP10)	CHH	21	−1.72		1.84
	AT3G04870	ZETA-CAROTENE DESATURASE (ZDS)	CHH	17	−0.35		
	AT3G27030	Transmembrane protein	CG	16	−0.93		0.76
	AT3G29185	BIOGENESIS FACTOR REQUIRED FOR ATP SYNTHASE 1 (BFA1)	CHG	28	−0.42		0.35
	AT3G55310	NAD(P)-binding Rossmann-fold superfamily protein	CG	29	−1.97		
	AT4G13110	BSD domain-containing protein	CG	18	−0.59		
	AT4G13160	Zein-binding protein (Protein of unknown function, DUF593)	CHH	20	−0.49		0.34
	AT5G17870	PLASTID-SPECIFIC 50S RIBOSOMAL PROTEIN 6 (PSRP6)	CHH	16	−0.34		
	AT1G54730	Major facilitator superfamily protein	CHG		0.64	25	−0.69
	AT3G28940	AIG2-like (avirulence induced gene) family protein	CHG		0.55	21	−1.06
	AT5G28610	LOW protein: ATP-dependent RNA helicase DRS1-like protein	CG			17	−2.15
	AT1G44970	PEROXIDASE9 (PRX9)	CG	20	4.13		
	AT1G51405	Myosin-like protein	CHH	22	1.61		
	AT1G66345	MITOCHONDRIAL INTRON SPLICING FACTOR 26 (MISF26)	CG	15	0.56		
	AT2G03980	GDSL-motif esterase/acyltransferase/lipase	CHH	18	1.07		
	AT2G43290	MULTICOPY SUPPRESSORS OF SNF4 DEFICIENCY IN YEAST 3 (MSS3)	CHH	16	1.01		−0.62
	AT3G28130	Nodulin MtN21-like transporter family protein (UMAMIT44)	CHG	16	0.79		
	AT3G29810	COBRA-LIKE PROTEIN 2 PRECURSOR (COBL2)	CHH	15	1.30		
	AT3G45160	Putative membrane lipoprotein	CHH	22	1.22		−0.73
	AT5G06195	Novel transcribed region	CG	36	1.74		
	AT5G22650	HISTONE DEACETYLASE 2B (HD2B; HDT2)	CG	27	0.43		
	AT5G46290	3-KETOACYL-ACYL CARRIER PROTEIN SYNTHASE I (KASI)	CG	20	0.41		−0.81
	AT5G53060	REGULATOR OF CBF GENE EXPRESSION 3 (RCF3)	CHH	−18	0.48		
	AT1G09530	PHYTOCHROME INTERACTING FACTOR 3 (PIF3)	CHG			−15	1.13
	AT2G20570	GBF’S PRO-RICH REGION-INTERACTING FACTOR 1 (GPRI1)	CHG			−22	2.35
	AT3G28500	60S acidic ribosomal protein family	CHG			−15	3.55
	AT3G45020	Ribosomal L18p/L5e family protein	CHG			−16	0.67
	AT5G17170	ENHANCER OF SOS3-1 (ENH1)	CHG			−16	1.06
	AT5G18065	HEAT-INDUCED TAS1 TARGET 3 (HTT3)	CHG			−17	0.76
	AT1G66340	ETHYLENE RESPONSE 1 (ETR1)	CHH	−21	−0.32		
	AT2G01680	Ankyrin repeat family protein	CHH	−19	−0.3		
	AT4G00755	F-box family protein	CHH	−27	−0.88		
	AT2G39100	RING/U-box superfamily protein	CG	−23	−0.62		0.63
	AT1G02730	CELLULOSE SYNTHASE-LIKE D5 (CSLD5)	CHH		1.45	−17	−0.83
	AT2G39320	Cysteine proteinases superfamily protein	CHG			−17	−2.85
	AT4G03460	Ankyrin repeat family protein	CHG			−20	−1.23
	AT4G10770	OLIGOPEPTIDE TRANSPORTER 7 (OPT7)	CHG		0.63	−21	−1.12
	AT4G13580	Disease resistance-responsive (dirigent-like protein) family protein	CHG		3.03	−17	−2.42
	AT5G03840	TERMINAL FLOWER 1 (TFL1)	CHH			−17	−1.68
	AT5G39110	RmlC-like cupins superfamily protein	CHG		1.68	−16	−1.30
	AT5G39670	CALMODULIN-LIKE 46 (CML46)	CHG		1.03	−18	−1.48

**Table 3:** Comparison of differentially accumulated miRNAs in *M. javanica*-induced Arabidopsis galls at early and mid-to-late infection stages. (**a**) miRNAs exclusively accumulated in galls at 3 dpi, and (**b**) in galls at 14 dpi as compared to control roots. (**c**) miRNAs differentially accumulated at both 3 and 14 dpi, showing either a common expression pattern or opposite regulation between the two gall stages. Red indicates upregulation, and blue indicates downregulation. Padj < 0.05.

(a)		
	FC in Galls	
miRNAs	At 3 dpi	At 14 dpi
ath-miR5643a	1.5	-
ath-miR156h	2.02	-
ath-miR775	2.13	-
ath-miR5655	2.46	-
ath-miR390b	2.7	-
ath-miR851-3p	2.94	-
ath-miR5657	3.47	-
ath-miR839	3.74	-
ath-miR156i	7.06	-
ath-miR156a	−2.47	-
ath-miR156c	−2.47	-
ath-miR156d	−2.5	-
ath-miR156e	−2.48	-
ath-miR156f	−2.48	-
ath-miR156j	−2.4	-
ath-miR159b	−1.52	-
ath-miR169d	−4.01	-
ath-miR169e	−4.01	-
ath-miR169f	−3.3	-
ath-miR169g-5p	−3.55	-
ath-miR169l	−3.55	-
ath-miR169h	−3.79	-
ath-miR169i	−3.67	-
ath-miR169j	−3.53	-
ath-miR169k	−3.79	-
ath-miR169l	−3.55	-
ath-miR169m	−4.03	-
ath-miR169n	−3.53	-
ath-miR399e	−3.02	-
ath-miR5635a	−1.78	-
ath-miR5635d	−1.98	-
ath-miR5637	−12.4	-
ath-miR5644	−4.09	-
ath-miR5645a	−1.6	-
ath-miR5645b	−1.6	-
ath-miR5645e	−1.59	-
ath-miR5645f	−1.59	-
ath-miR5648-3p	−8.58	-
ath-miR5653	−2.54	-
ath-miR780.2	−5.45	-
(**b**)		
	**FC in Galls**	
**miRNAs**	**At 3 dpi**	**At 14 dpi**
ath-miR397b	-	1.31
ath-miR158b	-	1.36
ath-miR5012	-	1.52
ath-miR164c-3p	-	1.81
ath-miR157a-3p	-	2.12
ath-miR859	-	2.3
ath-miR845a	-	2.53
ath-miR397a	-	2.94
ath-miR2111a-3p	-	4.76
ath-miR841a-5p	-	4.82
ath-miR771	-	−3.29
ath-miR777	-	−3.16
ath-miR172c	-	−3.12
ath-miR5648-5p	-	−2.65
ath-miR170-3p	-	−2.58
ath-miR399d	-	−2.34
ath-miR869.2	-	−2.34
ath-miR829-3p.1	-	−2.02
ath-miR829-5p	-	−2.01
ath-miR171c-5p	-	−1.99
ath-miR399f	-	−1.72
ath-miR399a	-	−1.69
ath-miR830-5p	-	−1.67
ath-miR822-3p	-	−1.62
ath-miR5654-5p	-	−1.6
ath-miR171b-3p	-	−1.55
ath-miR393b-3p	-	−1.52
ath-miR5650	-	−1.49
ath-miR829-3p.2	-	−1.27
(**c**)		
	**FC in Galls**	
**miRNAs**	**At 3 dpi**	**At 14 dpi**
ath-miR172e	−17.67	−3.34
ath-miR172a	−4.54	−2.76
ath-miR399b	−4.12	−1.70
ath-miR167d	−3.99	−1.62
ath-miR163	−3.66	−1.53
ath-miR857	−3.30	−2.03
ath-miR156b	−2.47	−1.48
ath-miR165a-5p	−1.65	−1.55
ath-miR165b	−1.67	−2.24
ath-miR166e	−1.55	−1.68
ath-miR166a-3p	−1.55	−1.54
ath-miR156g	−3.13	3.12
ath-miR319a/b	−4.88	1.33
ath-miR2111a-5p	−10.82	1.98
ath-miR390a	2.70	−1.38
ath-miR391	2.62	−1.82

## Data Availability

The datasets used or analyzed during the current study are available from the corresponding authors upon reasonable request. The complete raw sequencing data have been deposited in the GEO database under the accession number GSE320192 (RNA-seq 14 dpi) and GSE320193 (sRNA-seq 14 dpi).

## Supplementary Materials

The following supporting information can be downloaded at: https://www.mdpi.com/article/10.3390/ijms27104365/s1.
