# Supplementary material for: Integrative sRNA, DNA Methylation, and Transcriptomics Reveals Dynamic Epigenetic Reprogramming of Meloidogyne javanica-Induced Galls in Arabidopsis
Source: Int J Mol Sci. 2026 May 14;27(10):4365. doi: 10.3390/ijms27104365 (PMC13207400; doi:10.3390/ijms27104365)
Supplement: Supplementary file 1 [file ijms-27-04365-s001.zip › ijms-4246158-supplementary/ijms-4246158 Figure S1.pdf]

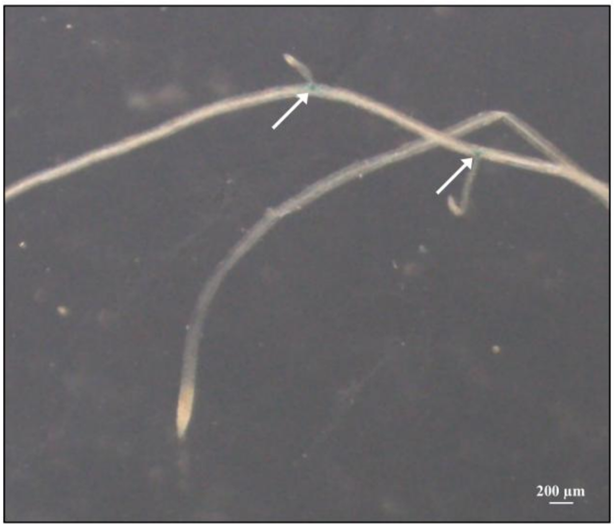

Figure S1. GUS assay of the reporter line *promiR2111a::GUS* in uninfected roots. GUS signal is only detected in the lateral root meristem area (white arrows). Scale bar: 200 µm.
